# Recombinant Human Interleukin-2 Corrects NK Cell Phenotype and Functional Activity in Patients with Post-COVID Syndrome

**DOI:** 10.3390/ph16040537

**Published:** 2023-04-03

**Authors:** Andrei A. Savchenko, Igor V. Kudryavtsev, Dmitry V. Isakov, Ivan S. Sadowski, Vasily D. Belenyuk, Alexandr G. Borisov

**Affiliations:** 1 Federal Research Center “Krasnoyarsk Science Center” of the Siberian Branch of the Russian Academy of Sciences, Scientific Research Institute of Medical Problems of the North, 660022 Krasnoyarsk, Russia; aasavchenko@yandex.ru (A.A.S.); sadovskii@rambler.ru (I.S.S.); dyh.88@mail.ru (V.D.B.); 2410454@mail.ru (A.G.B.); 2 Institute of Experimental Medicine, 197376 St. Petersburg, Russia; 3 School of Biomedicine, Far Eastern Federal University, 690922 Vladivostok, Russia; 4 Institute of Experimental Medicine, Pavlov First St. Petersburg State Medical University of the Russian Federation Ministry of Healthcare, 197022 St. Petersburg, Russia; issakovd71@gmail.com

**Keywords:** post-COVID syndrome, NK cell, recombinant human interleukin-2, immunorehabilitation, subset composition, functional activity

## Abstract

Post-COVID syndrome develops in 10–20% of people who have recovered from COVID-19 and it is characterized by impaired function of the nervous, cardiovascular, and immune systems. Previously, it was found that patients who recovered from infection with the SARS-CoV-2 virus had a decrease in the number and functional activity of NK cells. The aim of the study was to assess the effectiveness of recombinant human IL-2 (rhIL-2) administered to correct NK cell phenotype and functional activity in patients with post-COVID syndrome. Patients were examined after 3 months for acute COVID-19 of varying severity. The phenotype of the peripheral blood NK cells was studied by flow cytometry. It was found that disturbances in the cell subset composition in patients with post-COVID syndrome were characterized by low levels of mature (*p* = 0.001) and cytotoxic NK cells (*p* = 0.013), with increased release of immature NK cells (*p* = 0.023). Functional deficiency of NK cells in post-COVID syndrome was characterized by lowered cytotoxic activity due to the decreased count of CD57^+^ (*p* = 0.001) and CD8^+^ (*p* < 0.001) NK cells. In the treatment of patients with post-COVID syndrome with recombinant IL-2, peripheral blood NK cell count and functional potential were restored. In general, the effectiveness of using rhIL-2 in treatment of post-COVID syndrome has been proven in patients with low levels of NK cells.

## 1. Introduction

The novel coronavirus infection (COVID-19), caused by the virus SARS-CoV-2, is an acute infectious respiratory disease that can range in severity from mild (virtually asymptomatic), ending in self-recovery, to severe forms [1,2,3]. Patients with acute severe COVID-19 were characterized by damage to the lungs, development of multiple organ failure, and septic shock, resulting in an unfavorable disease outcome [1,3]. However, COVID-19 is not limited to the severity and outcome of the acute illness. Starting in the spring of 2020, some people (10–20%) who had recovered from coronavirus infection began to display short and long-term consequences manifested by a wide range of symptoms (headaches, fatigue, malaise, hyperthermia, shortness of breath, parosmia, muscle weakness, cognitive dysfunction, etc.) [4,5,6]. This condition is called “post-COVID syndrome”, which is characterized by a spectrum of persistent symptoms associated mainly with dysfunctions of the nervous, cardiovascular, and immune systems [7,8,9]. Moreover, in some cases, post-COVID syndrome can also be observed in patients due to asymptomatic or mild SARS-CoV-2 infection [10]. Nalbandian A. et al., (2021) classified the consequences of previous acute SARS-CoV-2 infection into sub-acute or persistent COVID-19 symptoms (up to 12 weeks from the initial acute episode) and chronic or post-COVID syndrome, where symptoms are observed for more than 12 weeks [11]. The Post-COVID-19 Functional Status Scale (PCFS) has been developed to rank post-COVID-19 patients for identifying prominent criteria and grouping into categories [12]. Accordingly, based on the characteristics of the course and symptoms of post-COVID syndrome, methods of patient rehabilitation are being developed [13,14].

The human immune system is actively involved in protecting against SARS-CoV-2 and developing effective and proper post-infection immunity that largely accounts for the healthy state after COVID-19 [15,16]. At the same time, in some cases, acute COVID-19 results in the formation of various disorders in the immune system that can persist even after recovery and can be involved in the underlying post-COVID syndrome. Thus, in patients recovering from COVID-19, persistent immune disorders were described as reduced NKT- and Vδ2 T-cell levels and increased count of low-density neutrophils, non-classical monocytes, hyperactivated CD8+ T-cells, along with an elevated level of pro-inflammatory cytokines [17]. At the same time, the association between a number of symptoms of post-COVID syndrome and a high serum IL-6 level was shown by Ganesh R. et al., (2022) [8]. In our previous study, it was found that individuals who underwent COVID-19 (SARS-CoV-2 negative PCR) showed an increased percentage of total T-lymphocytes (due to CD4+ T cell subsets) compared with controls, paralleled with a decreased absolute and relative level of B-cells (due to B1 and memory B2 B cell subsets) [18]. Townsend L. et al., (2021) showed that in COVID-19 convalescent patients, T-cell abnormalities persisted for more than 3 months, so that impaired T-cell immunity was more pronounced in older subjects [19].

NK cells are innate immune cells exerting cytotoxic activity [20,21]. Due to the fact that NK cell functions rely on developing both antitumor and antiviral protective mechanisms, the features of NK cell functionality in COVID-19 are of high interest. For instance, Deng X. et al., (2022) showed that the rate of SARS-CoV-2 elimination and the level of virus-specific antibody production correlated with the level of NK cell functional activity [22]. At the same time, they also noted that NK cell functional deficiency was revealed as increased viral susceptibility. Pituch-Noworolska A.M. (2022) showed that in patients with COVID-19, suppressed IFN-γ production by NK cells and their functional exhaustion were detected [23]. In convalescent patients, peripheral blood NK cell count may range widely (from reduced to increased relative to the normal level); however, even with a high NK cell level, their functional activity is reduced, which may be result in new viral infections, including COVID-19 [24,25,26]. In particular, a study assessing convalescent patients two weeks after COVID-19 revealed a low NK cell level [27]. Accordingly, it was noted that recovery of NK cell count and functional activity was contributed to developing effective antiviral immunity, including defense against SARS-CoV-2 [23,28].

It should be noted that recovery NK cell count and function in patients with post-COVID syndrome is important not only for developing but also for antitumor immunity. Therefore, the functional insufficiency of such cells resulting from COVID-19 may lead to rise in rate of oncology diseases. In this regard, it is very important to restore the number and function of NK cells during the immunorehabilitation of patients with post-COVID syndrome. It is known that interleukin-2 (IL-2) positively acts on NK cell functional activity [29,30,31].

Thus, the aim of the study was to assess an effectiveness of recombinant human IL-2 (rhIL-2) administered to correct NK cell phenotype and functional activity in patients with post-COVID syndrome.

## 2. Results

The clinical data analysis showed that patients with post-COVID syndrome (Post-COVID-19 Functional Status Scale score 1 to 3) mainly had general clinical manifestations and damage to the nervous system (Table 1). In addition, the respiratory and cardiovascular systems, and gastrointestinal tract were involved in the pathological process.

**Figure 1:**
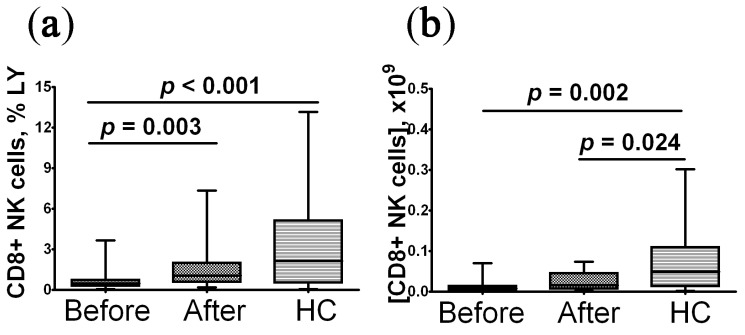
Alteration in relative and absolute level of peripheral blood NK cells in convalescent COVID-19 individuals before (*n* = 29) and after (*n* = 29) rhIL-2 treatment vs. the healthy control group (HC, *n* = 30). (**a**,**b**) – NK cells frequencies (% from total lymphocytes subset) and absolute numbers (number of cells in 1 litter), respectively. A comparison with the indicators of the control group was performed using the Mann–Whitney U test. A comparison of parameters in the patients before and after treatment with rhIL-2 was performed using the Wilcoxon matched pairs test.

When assessing the NK cell level in patients with post-COVID syndrome, it was found that before the onset of rhIL-2 treatment, the percentage and absolute level of NK cells was reduced compared to the control group; after treatment, the number of cells was restored to the level matching that of control magnitude (Figure 1).

Analyzing the NK cell level in patients with post-COVID syndrome by co-staining for CD16 and CD56 markers showed that prior to the onset of treatment, compared with the control group, the percentage of CD16^+^CD56^+^ and CD16^−^CD56^+^ cells was reduced, but the count of CD16^+^CD56^dim^ and CD16^−^CD56^dim^ NK cells was significantly increased (Table 2). After rhIL-2 treatment, the percentage of CD16^+^CD56^+^ cells increased by more than 3.5-fold compared with the baseline level, but their count remained significantly lower than in the control group. The level of CD16^−^CD56^+^ and CD16^−^CD56^dim^ cells barely changed after treatment. At the same time, the level of CD16^+^CD56^dim^ NK cells after rhIL-2 treatment increased by more than 4-fold compared to the baseline level.

We investigated the level CD57^+^ NK cells in patients with post-COVID syndrome before and after rhIL-2 treatment. It was found that before treatment vs. the control group, the percentage of CD57^+^ NK cells was significantly lowered (0.52% (0.31; 1.56) vs. 1.88% (1.05; 4.50) with *p* < 0.001), while after treatment it was increased (3.09% (2.04–5.09) vs. controls 1.88% (1.05; 4.50), *p* = 0.075). A similar time-dependent pattern was also noted for the absolute count of peripheral blood NK cells showing that in patients before vs. after therapy and the control group, it was significantly reduced (0.015 × 10^9^ cells/L (0.009–0.033) vs. 0.053 × 10^9^ cells/L (0.037–0.063) and 0.036 × 10^9^ cells/L (0.022–0.085), *p* = 0.004 and *p* = 0.011, respectively). The course of rhIL-2 therapy was accompanied by an increased level of NK cells to the range of the control group (0.053 × 10^9^ cells/L (0.037–0.063) vs. 0.036 × 10^9^ cells/L (0.022–0.085), *p* = 0.690).

The percentage of CD16^+^CD56^+^CD57^+^ NK cells in patients before and after treatment was significantly lower than in the control group, but after treatment their level was increased by 3.2-fold compared with the baseline (Table 3). The level of CD16^−^CD56^+^CD57^+^ NK cells from patients with post-COVID syndrome before treatment was lower than in the control group. After rhIL-2 treatment, their number did not change compared to baseline level and remained reduced compared to the control group. The percentage of CD16^+^CD56^dim^CD57^+^ and CD16^−^CD56^dim^CD57^+^ NK cells before treatment matched that of the control group. After treatment, the level of CD16^+^CD56^dim^CD57^+^ NK cells significantly increased both compared to baseline and control magnitude, whereas the count of CD16^−^CD56^dim^CD57^+^ NK cells remained unchanged.

Of note, the CD57 expression level (shown in arbitrary MFI units) on total NK cells and most of its subsets in patients with post-COVID syndrome before and after rhIL-2 treatment did not differ from the control group, except for only CD16^−^CD56^+^ NK cells. The expression level of CD57 on this NK cell subset in patients before treatment was significantly reduced compared to control group (0.32 (0.24; 0.38) vs. 0.36 (0.32; 0.45), *p* = 0.029), whereas after rhIL-2 treatment, CD57 MFI was restored to the control level (0.36 (0.27; 0.44) vs. 0.36 (0.32; 0.45), *p* = 0.374).

Additionally, depending on the surface CD57 and CD62L marker expression, the total pool of NK cells can be divided into four major subsets that differed not only in the migratory potential to lymphoid (CD62L^+^ cells) or peripheral (CD62L^–^ cells) tissues, but also in the capacity of cytolytic properties against target cells due to accumulated cytosolic perforin and granzymes (CD57^+^ cells) [32]. It was found that the percentage of CD57^+^ NK cells expressing CD62L in patients with post-COVID syndrome before rhIL-2 treatment was significantly lower than in the control group, but after treatment, it increased compared to the baseline magnitude to the level of the control range (Table 4).

**Figure 2:**
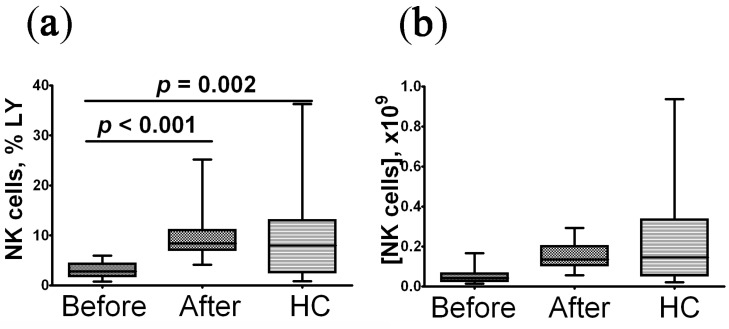
Imbalanced relative and absolute levels of peripheral blood CD8^+^ NK cells in convalescent COVID-19 individuals before (*n* = 29) and after (*n* = 29) recombinant human IL-2 treatment vs. the healthy control group (HC, *n* = 30). (**a**,**b**)—CD8^+^ NK cells frequencies (% from total lymphocytes subset) and absolute numbers (number of cells in 1 litter), respectively. The statistical analysis was performed with the Mann–Whitney U test.

The percentage and absolute count of blood CD8^+^NK from patients with post-COVID syndrome before treatment were significantly reduced compared to the control group (Figure 2). After rhIL-2 treatment, the relative level of CD8^+^NK cells was restored to the control level, whereas changes in their absolute count were not statistically significant.

The percentage of CD16^+^CD56^+^CD8^+^ cells in patients examined before rhIL-2 treatment was significantly reduced compared to the control group, whereas after treatment, their count was increased by 2-fold compared to the baseline level, but remained low compared to the control group (Table 5). At the same time, the CD16^−^CD56^+^CD8^+^ NK cell count in patients with post-COVID syndrome did not change during immunorehabilitation and was significantly lower than in the control group throughout the entire experiment. The level of CD16^+^CD56^dim^CD8^+^ NK cells before treatment was more than 5-fold higher than in the control group. After treatment, the count of this cell type increased, both compared to baseline level and control group. At the same time, CD16^−^CD56^dim^CD8^+^ NK cells in patients with post-COVID syndrome before and after rhIL-2 treatment corresponded to the level of the control group.

While examining CD94^+^ NK cell count, it was found that in patients with post-COVID syndrome before immunorehabilitation, the percentage of CD56^+^CD94^+^ NK cells was significantly reduced compared to the control level, whereas for CD56^dim^CD94^+^ NK cells, it matched to that of the control group (Table 6). After the immunorehabilitation, the count of CD56^+^CD94^+^ NK cells increased by more than 2-fold; however, while remaining at a reduced level compared to the control magnitude. The relative number of CD56^dim^CD94^+^ NK cells after rhIL-2 therapy also increased and became significantly higher than in the control group. The level of CD94 expression on CD56^+^ NK cells in patients with post-COVID syndrome was increased compared to control group even before the onset of immunorehabilitation and remained at this level after rhIL-2 treatment, whereas expression level of this marker on CD56^dim^ NK cells in examined patients before and after immunorehabilitation corresponded to that of the control group.

Limitations of the study. The sample size in our study was limited due to the availability and willingness of patients to donate whole blood for the analyses before and after treatment. In addition, the study treatment design was open-label.

## 3. Discussion

NK cells are a heterogeneous population displaying natural cytolytic activity against virus-infected and malignant cells capable of producing cytokines and chemokines [20,21,33]. We assessed NK cell subset composition by staining for surface markers CD16 and CD56. CD16 is a low-affinity immunoglobulin G type III receptor (FcγRIII), which mediates the mechanism of cellular cytotoxicity [34]. The CD56 marker (NCAM, Leu-19, NKH-1) is a glycoprotein belonging to the immunoglobulin superfamily, which is involved in establishing intercellular contacts [35,36]. At the same time, mature NK cells are defined by active expression of CD16 and CD56 markers. CD16^−^CD56^+^ NK cells exhibit cytotoxic activity [37,38]. Of great interest are CD16^+^CD56^dim^ and CD16^−^CD56^dim^ NK cells. It has been shown that CD16^+^CD56^dim^ NK cells show increased cytotoxicity, which are found in peripheral blood, and are also localized in the lungs as well as inflammation foci (due to the unique profile of chemokine receptors expression), which allows them to be characterized as pro-inflammatory NK cells [39]. At the same time, the CD16^−^CD56^dim^ NK cells are predominant in tumors (possibly with regulatory activity), which are characterized as less mature NK cells compared to the CD16^+^CD56^dim^ subset [39,40].

Our study revealed that before the onset of rhIL-2 therapy, the patients with post-COVID syndrome had a decreased percentage of mature and cytokine-producing NK cells but an increased level of CD56^dim^ immature cells. It should be noted that such changes in the NK cell subset composition emerge along with a decreased percentage and absolute count of total NK cells. After immunorehabilitation, the quantity of total NK cells is fully restored to control magnitude. The level of mature (CD16^+^CD56^+^) NK cells is elevated by 3-fold compared to baseline level, but remains significantly reduced relative to control values. The low level of cytokine-producing NK cells 20 and elevated percentage of CD16^−/+^CD56^dim^ cells remained virtually intact after rhIL-2 treatment. At the same time, the quantity of cytotoxic cells increased more than by 4-fold. Overall, it can be concluded that in patients with post-COVID syndrome with quantitative recovery to the control level of total NK cells, altered subset composition remains.

We investigated the distribution of the CD57 marker on NK cell subsets in patients with post-COVID syndrome. CD57 antigen (HNK-1, NK-1, and Leu-7) is a sulfated glucuronic acid oligosaccharide, which is expressed on various proteins, lipids, and chondroitin sulfate proteoglycans, defined as a marker of terminally differentiated NK cells with increased functional activity [41,42]. In our study, patients with post-COVID syndrome had a reduced absolute and relative level of CD57 expressing NK cells before immunorehabilitation. The level of CD16^+^CD56^+^CD57^+^ and CD16^−^CD56^+^CD57^+^ NK cells was significantly reduced compared to the control group, whereas that of for the CD16^−/+^CD56^dim^CD57^+^ subset was equal to the control group. At the same time, a marked decrease in expression (by MFI level) of the surface CD57 marker on cytokine-producing NK cells is revealed. Kared H. et al., (2016) reviewed that CD16^+^CD57^+^ NK cells display the maximum cytotoxic effect, whereas CD57^+^ NK cells, in general, contribute to dendritic cell maturation, and additional stimulation of CD57^+^CD56^dim^CD16^+^ NK cells with pro-inflammatory cytokines significantly increased IFN-γ production and secretion along with exerted antibody-dependent cytotoxicity [41]. Consequently, the functional potential of NK cells in patients with post-COVID syndrome was significantly reduced. After rhIL-2 treatment, the quantity of total blood NK cells was restored in the patients examined. The level of CD16^+^CD56^+^CD57^+^ NK cells is increased by more than 3-fold, although, at the same time, they remain lower than in the control group. The CD57 expression level on cytokine-producing NK cells was restored, but their quantity remains unaltered. The level of CD16^+^CD56^dim^CD57^+^ cells significantly increased both compared to baseline and control magnitude. Thus, immunorehabilitation of patients with post-COVID syndrome treated with rhIL-2 leads to recovery of CD57^+^ NK cell quantity. The restored expression of this marker on cytokine-producing NK cells, as well as an increased level of CD57^+^ cytotoxic NK cells, also reflects the rise in functional activity of NK cells. It should be noted that profound disturbances in the post-COVID period are also observed in the subset composition of another population of cytolytic cells, CD8^+^ T-lymphocytes. Thus, it was shown that 1–3 months after acute SARS-CoV-2 infection, the relative and absolute level of peripheral blood “I” as well as central memory CD8^+^ T cells increases, whereas the TEMRA subset decreased compared to the control level [43]. In another study, it was shown that within the interval covering from 2–4 weeks to 2–3 months of PSO, the level of “naïve” and “transient” CD8^+^ T-memory cells decreased, whereas the percentage of peripheral blood central and effector memory as well as mature effector CD8^+^ T-cells increased [44]. Long-term disturbances in maturation and differentiation of NK cells and CD8^+^ T cells, the expression of surface inhibitory receptors or “cellular aging” markers, as well as low efficiency of target cell destruction can reduce the efficacy of antitumor and antiviral immunity [45,46].

The CD62L marker is an L-selectin that accounts for immune cell migration to peripheral lymphoid organs because its cognate ligands are presented by GlyCAM-1 (glycosylation-dependent cell adhesion molecule-1), MadCAM-1 (mucosal addressin cell adhesion molecule-1), and CD34 (an endothelial cell protein) [47,48]. We have found that before the onset of immune rehabilitation in patients with post-COVID syndrome, the level of CD57^+/−^CD62L^+^NK cells were significantly lower than in the control group allowing us to conclude low lymphocyte migratory activity in the examined patients. After rhIL-2 treatment, the count of CD62L^+^ NK cells increased to the level of the control range, which characterizes recovery of their migratory activity.

In patients with post-COVID syndrome, we also examined the level of peripheral blood CD8^+^NK cells. It is known that about 40% of NK cells can express the CD8 α/α-homodimeric receptor, which accounts for their high cytotoxic activity [49,50]. It is also suggested that the interaction between surface NK cell CD8αα receptors and HLA-I family molecules may cause increased secretion of cytokines such as TNF-α and IFN-γ [51]. We have found that in patients with post-COVID syndrome, the absolute and relative level of peripheral blood CD8^+^NK cells are significantly reduced. At the same time, the expression level of the CD8 receptor on the cells also declines. While examining the expression of this molecule on various NK cell subsets, it was found that in patients with post-COVID syndrome, the level of mature (CD16^+^CD56^+^) and cytotoxic (CD16^−^CD56^+^) CD8 expressing NK cells was reduced compared to the control group. At the same time, the level of pro-inflammatory NK cells (CD16^+^CD56^dim^) with CD8 co-expression was significantly higher than in the control group. In addition to the reorganized NK cell subset composition in patients with post-COVID syndrome, we also found that mature and cytotoxic NK cells express this marker at a lower level. After treatment, the percentage of CD8^+^NK cells recovered to that of the control group, whereas the absolute count was 2-fold higher but remained below that of found in healthy subjects. Administration of rhIL-2 was able to elevate the level of mature CD8^+^NK cells by 2-fold, whereas for CD8^+^ NK cells it was unchanged. At the same time, the count of pro-inflammatory CD8^+^ NK cells in patients with post-COVID syndrome increased by more than 2-fold after immune rehabilitation. The percentage of CD16^−^CD56^dim^CD8^+^ NK cells in patients with post-COVID syndrome did not respond to cytokine therapy, both before and after immune rehabilitation, and matched that of the control group.

CD94 is a type II integral membrane protein belonging to the family of Ca^2+^-dependent lectins (type C) able to bind to five different members of the NKG2 family (NKG2A, B, C, E, and H). It has been proven that CD94/NKG2 receptors show specificity for HLA-E (a non-classical MHC class I molecule) [52,53]. At the same time, CD94/NKG2A and CD94/NKG2B heterodimers are a part of the inhibitory NK-cell receptors, and the CD94-NKG2C belongs to an activating receptor. In the patients with post-COVID syndrome examined by us, before immune rehabilitation, the percentage of CD56^+^CD94^+^NK cells was reduced by more than 30-fold compared to the control group, whereas the CD94 expression level on such cells was 1.8-fold higher than in healthy individuals. At the same time, the quantity of CD56^dim^CD94^+^NK cells in such patients corresponded to the control group as well as its expression level. After rhIL-2 treatment, the level of CD56^+^CD94^+^NK cells was increased by more than 2-fold, although it still remained below the control level. The surface CD94 expression level, however, did not change. At the same time, the quantity of CD56^dim^CD94^+^ NK cells after cytokine therapy in patients with post-COVID syndrome significantly increased, but without changes in the CD94 expression level. It should be noted that, based on the specification (clone R34.34, cat. IM1980U, Beckman Coulter, Indianapolis, IN, USA), the CD94 monoclonal antibody binds with greater specificity to the NKG2A receptor. Accordingly, a markedly increased quantity of CD56^dim^CD94^+^ NK cells can be associated with the release of immature cells into the blood, among which there may also be regulatory NK cells.

IL-2 is a powerful inducer of NK cell proliferation also affecting cell phenotype and stimulating the production of lytic molecules (such as perforin and granzyme B) [53,54,55,56]. In particular, the study by Sharma R. and Das A. (2018) showed that IL-2 stimulation led to an enlarged NK cell population and upregulated expression level of activation markers [55]. During the rhIL-2 immune rehabilitation of patients with post-COVID syndrome, recovery of the absolute and relative level of peripheral blood NK cells were also achieved. At the same time, disturbances in the subset composition were revealed as a decreased count of mature and cytotoxic NK cells along with an elevated level of pro-inflammatory and immature NK cells. The count of CD57^+^NK cells was completely restored. It is known that CD57 marker expression is associated with the accumulation of perforin and granzymes in cytotoxic cells, allowing us to use this marker to quantify cells with high cytolytic potential [57]. After the immunorehabilitation of patients with post-COVID syndrome, the level of CD8^+^NK cells, also exerting high cytotoxic activity as well as migration potential of NK cells (by the CD62L marker), was restored. In general, recombinant human IL-2 in immunorehabilitation of patients with post-COVID syndrome allowed significantly stimulation of NK cell functional potential, which is undoubtedly crucial for preventing viral infections and cancer immunosurveillance.

## 4. Materials and Methods

### 4.1. Patients

An observational therapeutic prospective clinical study was conducted with patients undergoing rehabilitation at the Scientific Research Institute of Medical Problems of the North (Federal Research Center “Krasnoyarsk Science Center” of the Siberian Branch of the Russian Academy of Sciences), 3 months after the onset of acute COVID-19. We enrolled 29 patients (15 females and 14 males, aged 40–65 years) who had recovered after COVID-19 of varying severity. Clinical and demographic characteristics of the patients were described based on discharge reports and extracts from outpatient records (Table 7).

The inclusion criteria were: prior acute COVID-19 (severity ranging from 3 to 7 score according to the WHO criteria), clinical manifestations of post-COVID-19 syndrome (post-COVID-19 Functional Status Scale 1–3), signed informed consent in accordance with the Helsinki declaration of the World Association “Ethical principles of scientific medical research involving humans” and the Guidelines of Clinical Practice in the Russian Federation, NK cell absolute count less than 0.07 × 10^9^ cells/L, and/or relative number less than 5% out of lymphocyte population. The WHO Clinical Progression Scale and the Post-COVID-19 Functional Status Scale were assessed using standard methods [2,12]. Patients with autoimmune diseases, heart and pulmonary heart failure, cancer, pregnancy and lactation, yeast hypersensitivity, IL-2 or any drug component use in their history, as well as patients with decompensated liver and kidney failure were excluded from the study.

To correct immunological parameters and increase NK cell count by applying immunocorrective therapy, all patients with post-COVID syndrome received recombinant human IL-2 (hrIL-2) at a dose of 0.5 mg (equivalent to 500,000 IU) subcutaneously, three injections with an interval of 48 h, along with basic therapy (detoxification, metabolic, symptomatic therapy). Immunological studies were performed twice before and after hrIL-2 therapy. As a control group, 35 age-matched volunteers were examined. The study protocol was approved by the Ethics Committees of Scientific Research Institute of Medical Problems of the North (Federal Research Center “Krasnoyarsk Science Center” of the Siberian Branch of the Russian Academy of Sciences) (protocol 02/2022 dated of 17 February 2022).

### 4.2. NK Cell Phenotype Assessed by Flow Cytometry

Venous blood samples were collected in K_3_EDTA vacuum tubes (Becton Dickinson, Franklin Lakes, NJ, USA). The study of NK cell phenotype was carried out no later than 2 h after blood sampling. Preparation of blood samples and flow cytometer setup were performed in accordance with the recommendations of the antibody manufacturers. The following panel of monoclonal antibodies (MATs) conjugated to various fluorochromes (all MATs manufactured by Beckman Coulter, Indianapolis, IN, USA) was used to study NK cell phenotype (Table 8). It contained the following MATs: FITC-labeled mouse anti-human CD57 (clone NC1, cat. IM0466U, Beckman Coulter, Indianapolis, IN, USA), PE-labeled mouse anti-human CD94 (clone R34.34, cat. IM1980U, Beckman Coulter, Indianapolis, IN, USA), ECD-labeled mouse anti-human CD62L (clone DREG5, cat. IM2276, Beckman Coulter, Indianapolis, IN, USA), PC5.5-labelled mouse anti-human CD56 (clone N901, cat. A07789, Beckman Coulter, Indianapolis, IN, USA), PC7-labelled mouse anti-human CD16 (clone 3G8, cat. 6607118, Beckman Coulter, Indianapolis, IN, USA), APC-labelled mouse anti-human CD8 (clone B9. 11, cat. IM2469, Beckman Coulter, Indianapolis, IN, USA), A-A700-labelled mouse anti-human CD3 (clone UCHT1, cat. B10823, Beckman Coulter, Indianapolis, IN, USA), and A-A750-labelled mouse anti-human CD45 (clone J33, cat. A79392, Beckman Coulter, Indianapolis, IN, USA). These MAb cocktails were used to stain 100 µL of a blood sample in accordance with the manufacturer’s recommendations. Erythrocytes were removed from the samples using no-wash technology with VersaLyse Lysing Solution (Beckman Coulter, Indianapolis, IN, USA, cat. A09777), and 25 µL of IOTest 3 Fixative Solution (Beckman Coulter, Inc., Indianapolis, IN, USA, cat. A07800) was added to 975 µL of sample. For data analysis, there was used Navios™ flow cytometer (Beckman Coulter, Indianapolis, IN, USA) at Krasnoyarsk Regional Center of Research Equipment of Federal Research Center Krasnoyarsk Science Center SB RAS. At least 50,000 lymphocytes were analyzed in each sample.

### 4.3. Statistical Analysis

**Figure 3:**
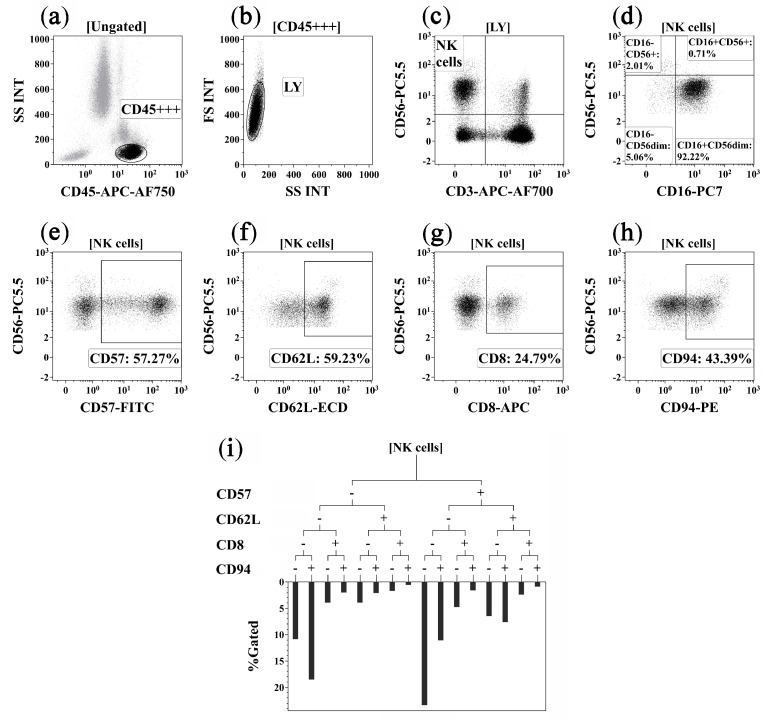
Flow cytometry immunophenotyping gating strategy for NK cell subsets. (**a**) Total lymphocyte subset identification based on side scatter and bright CD45 expression; (**b**) discrimination between single lymphocytes, doublets, and cell debris; (**c**) total NK cell subset was detected as CD3^−^CD56^+^; (**d**) co-expression of CD16 and CD56 on main NK cell subsets, including CD16^+^CD56^+^, CD16^−^CD56^+^, CD16^+^CD56^dim^, and CD16^−^CD56^dim^ NK cells; (**e**–**h**) expression of CD57, CD62L, CD8, and CD94 by NK cells, these gates were used as branches for the hierarchical tree histogram; (**i**) hierarchical tree histogram was gated on total NK cell subsets as an example. The frequency histogram below the trees indicated the relative proportion of cells with different patterns of CD57, CD62L, CD8, and CD94 co-expression within total NK cells.

Flow cytometry data were processed using Navios Software v.1.2 and Kaluza™ v.2.2 (Beckman Coulter, Indianapolis, IN, USA). The ‘Gaiting strategy’ is shown on Figure 3.

The data sample was assessed by calculating the median (Me) and interquartile ranges (IQR), along with the 25th and 75th percentiles. Qualitative variables of clinical parameters were presented in absolute values and percentages (*n* (%)). The significance of differences for quantitative parameters from unrelated samples was assessed using the nonparametric Mann–Whitney U test. The significance of differences for quantitative parameters from patients with post-COVID syndrome before and after rhIL-2 treatment was assessed by the Wilcoxon matched pairs test. Comparison of qualitative variables was carried out using exact χ^2^. Statistical analysis was carried out using the Statistica 8.0 software package (StatSoft, Tulsa, OK, USA, 2007).

## 5. Conclusions

During acute SARS-CoV-2 infection, the virus hampers the proper type 1 immune response by impairing the functionality of Th1 cells, CD8^+^ T cells, and NK cells [15,16]. Similarly, after acute COVID-19, many patients faced post-COVID-19 syndrome, which influenced the functioning of various immune organs and cells in acute COVID-19 convalescent patients [14,17]. Currently, recommendations for the rehabilitation of the post-COVID condition already exist [58,59]; however, there is still a lack of evidence on the effectiveness of these interventions in individuals with post-acute COVID-19 syndrome. Our data indicates that, in patients with post-COVID syndrome, the count and functional activity of peripheral blood NK cells were reduced. Disturbances in the cell subset composition were characterized by low levels of mature and cytotoxic NK cells, with increased release of immature NK cells. Functional deficiency of NK cells in post-COVID syndrome was characterized by lowered cytotoxic activity due to a decreased count of CD57^+^ and CD8^+^ NK cells. In addition, in patients with post-COVID syndrome, the migration potential of NK cells was reduced. In the treatment of patients with post-COVID syndrome with recombinant IL-2, the peripheral blood NK cell count and functional potential were restored. It should be noted that cytokine therapy did not lead to the recovery of the NK cell subset composition, and the level of NKG2A^+^CD56^dim^ NK cells, including regulatory cells, also was increased in patients. In general, the effectiveness of using rhIL-2 in the treatment of post-COVID syndrome has been proven in patients with low levels of NK cells. In the future, it will be important to assess the effect of rhIL-2 treatment on other immune cells that take part in type 1 immunity. Accordingly, it is additionally necessary to evaluate the effectiveness of such immunotherapy in post-COVID syndrome for restoring other arms of the immune system.

## Figures and Tables

**Table 1:** Clinical manifestations in patients with post-COVID syndrome in the dynamics of immunorehabilitation using rhIL-2.

Parameters	Before Treatment	After Treatment
Feeling sick after exercise, *n*/%	29/100%	1/3.45%
Fatigue, *n*/%	29/100%	2/6.90%
Headache, *n*/%	26/89.66%	1/3.45%
Subfebrile temperature, *n*/%	18/62.07%	0
Memory problems, inability to concentrate, *n*/%	22/75.86%	1/3.45%
Cognitive dysfunction, *n*/%	9/31.03%	0
Sleep disturbance, *n*/%	7/24.14%	0
Muscle pain, *n*/%	1/3.45%	0
Joint pain, *n*/%	6/20.69%	1/3.45%
Cough, *n*/%	10/34.48%	0
Dyspnea, *n*/%	3/10.34%	0
Tachycardia, *n*/%	3/10.34%	0
Skin rash, *n*/%	4/13.79%	0
Diarrhea, *n*/%	1/3.45%	0
Leukocytes, 10^9^/L ME (IQR)	5.69 (5.00–6.75)	5.25 (4.23–6.40)
Granulocytes, % ME (IQR)	56.0 (43.0–66.8)	57.1 (43.0–72.4)
Granulocytes, 10^9^/L ME (IQR)	2.48 (4.50–4.14)	3.00 (1.57–5.00)
Monocytes, % ME (IQR)	7.0 (6.8–9.3)	7.4 (5.0–9.5)
Monocytes, 10^9^/L ME (IQR)	0.38 (0.31–0.59)	0.38 (0.24–0.48)
Lymphocytes, % ME (IQR)	33.7 (26.7–43.4)	30.0 (20.9–40.3)
Lymphocytes, 10^9^/L ME (IQR)	2.36 (1.92–3.03)	1.62 (1.21–2.20)

Quantitative data are presented by the median and interquartile ranges (median (IQR)).

**Table 2:** Major peripheral blood NK cell subsets (%) in convalescent COVID-19 individuals before and after recombinant human IL-2 treatment (Me (IQR)).

NK Cell Subset	Healthy Controls	Before Treatment	After Treatment
CD16^−^CD56^+^	0.80 (0.15–2.50)	0.13 (0.08–0.79) *p*_1_ = 0.013	0.29 (0.11–0.55) *p*_1_ = 0.049
CD16^+^CD56^+^	5.88 (0.23–11.70)	0.21 (0.13–0.39) *p*_1_ = 0.001	0.74 (0.44–1.23) *p*_2_ < 0.013
CD16^−^CD56^dim^	0.46 (0.10–0.88)	0.91 (0.30–1.77) *p*_1_ = 0.023	1.33 (0.65–2.12) *p*_1_ = 0.001
CD16^+^CD56^dim^	0.69 (0.07–1.33)	0.97 (0.46–1.71)	4.19 (2.78–7.36) *p*_1,2_ < 0.001

The quantitative data (% NK cell subsets within total lymphocytes population) are presented as median and quartile ranges (Me (IQR)); *p*_1_—statistically significant differences versus controls; *p*_2_—statistically significant differences in patients before and after treatment. A comparison with the indicators of the control group was performed using the Mann–Whitney U test. Comparison of parameters in the patients before and after treatment with rhIL-2 was performed using the Wilcoxon matched pairs test.

**Table 3:** CD57 expressed on diverse NK cell subsets (%) in convalescent COVID-19 individuals before and after recombinant human IL-2 treatment (Me (IQR)).

NK Cell Subset	Healthy Controls	Before Treatment	After Treatment
CD16^−^CD56^+^	0.24 (0.03–0.95)	0.03 (0.01–0.21)	0.05 (0.01–0.08) *p*_1_ = 0.008
CD16^+^CD56^+^	3.30 (0.12–5.35)	0.13 (0.09–0.25) *p*_1_ < 0.001	0.42 (0.23–0.67) *p*_2_ < 0.001
CD16^−^CD56^dim^	0.09 (0.03–0.13)	0.11 (0.04–0.85)	0.23 (0.05–0.47)
CD16^+^CD56^dim^	0.51 (0.06–1.02)	0.36 (0.18–1.16)	1.88 (1.35–4.22) *p*_1,2_ < 0.001

The quantitative data (% of CD57-expressing cells within total lymphocytes) are presented as median and quartile ranges (Me (IQR)); *p*_1_—statistically significant differences versus controls; *p*_2_—statistically significant differences in patients before and after treatment. A comparison with the indicators of the control group was performed using the Mann–Whitney U test. A comparison of parameters in the patients before and after treatment with rhIL-2 was performed using the Wilcoxon matched pairs test.

**Table 4:** CD57 and CD62L co-expressing NK cell subsets (%) in convalescent COVID-19 individuals before and after recombinant human IL-2 treatment (Me (IQR)).

NK Cell Subset	Healthy Controls	Before Treatment	After Treatment
CD62L^+^CD57^−^	1.74 (0.85–4.11)	0.38 (0.23–1.52) *p*_1_ = 0.001	2.62 (1.97–4.29) *p*_2_ < 0.001
CD62L^+^CD57^+^	0.26 (0.14–0.63)	0.11 (0.08–0.19) *p*_1_ = 0.009	0.62 (0.16–0.90) *p*_2_ < 0.001
CD62L^−^CD57^−^	2.67 (0.32–4.90)	0.53 (0.33–0.92) *p*_1_ = 0.001	2.18 (1.42–3.10) *p*_2_ < 0.001
CD62L^−^CD57^+^	1.09 (0.65–1.66)	0.85 (0.53–1.64)	1.60 (0.99–3.28) *p*_2_ = 0.003

The quantitative data (% of NK cell subsets within total lymphocytes) are presented as median and quartile ranges (Me (IQR)); *p*_1_—statistically significant differences versus controls; *p*_2_—statistically significant differences in patients before and after treatment. A comparison with the indicators of the control group was performed using the Mann–Whitney U test. A comparison of parameters in the patients before and after treatment with rhIL-2 was performed using the Wilcoxon matched pairs test.

**Table 5:** CD8 expression by diverse NK cell subsets (%) in convalescent COVID-19 individuals before and after recombinant human IL-2 treatment (Me (IQR)).

NK Cell Subset	Healthy Controls	Before Treatment	After Treatment
CD16^−^CD56^+^	0.24 (0.05–0.80)	0.05 (0.02–0.11) *p*_1_ = 0.014	0.04 (0.01–0.05) *p*_1_ = 0.001
CD16^+^CD56^+^	1.48 (0.12–4.40)	0.05 (0.04–0.09) *p*_1_ < 0.001	0.11 (0.06–0.33) *p*_1_ = 0.006 *p*_2_ = 0.004
CD16^−^CD56^dim^	0.07 (0.03–0.22)	0.06 (0.01–0.23)	0.08 (0.01–0.25)
CD16^+^CD56^dim^	0.24 (0.04–0.76)	0.21 (0.07–0.43)	0.50 (0.29–1.25) *p*_1_ = 0.022 *p*_2_ = 0.002

The quantitative data (% of CD8-expressing cells within total lymphocytes) are presented as median and quartile ranges (Me (IQR)); *p*_1_—statistically significant differences versus controls; *p*_2_—statistically significant differences in patients before and after treatment. A comparison with the indicators of the control group was performed using the Mann–Whitney U test. A comparison of parameters in the patients before and after treatment with rhIL-2 was performed using the Wilcoxon matched pairs test.

**Table 6:** CD56 and CD94 co-expressing NK cell subsets in convalescent COVID-19 individuals before and after recombinant human IL-2 treatment (Me (IQR)).

NK Cell Subset	Healthy Controls	Before Treatment	After Treatment
CD56^+^CD94^−^	1.03 (0.06–2.91)	0.04 (0.02–0.64) *p*_1_ = 0.001	0.09 (0.04–0.15) *p*_1_ = 0.007
CD56^+^CD94^+^	5.25 (0.13–8.22)	0.20 (0.11–0.36) *p*_1_ = 0.001	0.48 (0.18–0.75) *p*_1_ = 0.047 *p*_2_ = 0.004
CD56^dim^CD94^−^	0.50 (0.36–1.07)	1.01 (0.38–1.91)	2.57 (1.37–3.70) *p*_1_ < 0.001 *p*_2_ = 0.001
CD56^dim^CD94^+^	0.69 (0.17–1.42)	1.25 (0.53–1.55)	3.60 (2.47–5.89) *p*_1,2_ < 0.001

The quantitative data (% of NK cell subsets within total lymphocytes) are presented as median and quartile ranges (Me (IQR)); *p*_1_—statistically significant differences versus controls; *p*_2_—statistically significant differences in patients before and after treatment. A comparison with the indicators of the control group was performed using the Mann–Whitney U test. A comparison of parameters in the patients before and after treatment with rhIL-2 was performed using the Wilcoxon matched pairs test.

**Table 7:** Clinical and demographic characteristics of examined patients with post-COVID syndrome.

Characteristics	Indicators
Females, *n*/%	15/51.72%
Males, *n*/%	14/48.28%
Average age, years	57.0
Age groups, *n*/%	
<45	8/27.59%
45–59	15/51.72%
≥60	6/20.69%
WHO Clinical Progression Scale, *n*/%	
1−3	13/44.83%
4−5	12/41.38%
6−7	4/13.79%
Symptoms, *n*/%	
Fever	29/100%
Other symptoms of intoxication	29/100%
Cough	24/82.76%
Other respiratory symptoms	29/100%
Respiratory failure with respiratory support	4/13.79%
Respiratory failure with intubation and mechanical ventilation	2/6.90%
Anosmia	5/17.24%
Gastrointestinal tract injury	11/37.93
Diarrhea	3/10.34
Biomarkers, Me (IQR)	
Leukocytes, 10^9^ cells/L	5.38 (4.21–7.53)
Lymphocytes, 10^9^ cells/L	0.80 (0.47–1.53)
C-reactive protein, mg/L	62.4 (25.2–98.5)
D-dimers, ng/mL	653.0 (435.7–904.8)
Lactate dehydrogenase activity, ME/L	638 (417–811)
Complications, *n*/%	
Pneumonia, including	22/75.86%
CT scan findings-CT1	12/50.0%
CT scan findings-CT2	8/40.91%
CT scan findings-CT3	2/9.09%
Systemic inflammatory response syndrome	29/100%
Damage to the cardiovascular system	3/10.34%
Acute renal failure	1/3.45%
Acute liver failure	3/10.34%
Depression, anxiety	6/20.69%
Several complications	23/79.31%
Accompanying illnesses, *n*/%, including	
Hypertonic disease	23/79.31%
Other cardiovascular diseases	9/31.03%
Cerebrovascular insufficiency	6/20.69%
Diabetes	2/6.9%
Chronic lung disease	1/3.45
Chronic liver disease	3/10.34
Obesity	9/31.03%
Other diseases	5/17.24%
Several diseases	27/93.10%
Post-COVID-19 Functional Status Scale, *n*/%	
1	9/31.03%
2	14/48.28%
3	6/20.69%

**Table 8:** Maim characteristics of antibodies used for flow cytometry.

No	CDs	Fluo.	Clone	Cat	Dilution	NK Cell Subset
1	CD57	FITC	NC1	IM0466U	1:4	Effector NK cells
2	CD94	PE	R34.34	IM1980U	1:2	Immature NK cells
3	CD62L	ECD	DREG5	IM2276	1:4	Immature NK cells
4	CD56	PC5.5	N901	A07789	1:2	Lineage NK cells marker
5	CD16	PC7	3G8	6607118	1:4	NK cell subsets
6	CD8	APC	B9.11	IM2469	1:8	NK cell subset marker
7	CD3	APC-A700	UCHT1	B10823	1:4	T cell exclusion
8	CD45	APC-A750	J33	A79392	1:4	Lineage lymphocytes marker, cell debris exclusion

## Data Availability

Data is contained within the article.
